# Effect of CT imaging on the accuracy of the finite element modelling in bone

**DOI:** 10.1186/s41747-020-00180-3

**Published:** 2020-09-01

**Authors:** Emir Benca, Morteza Amini, Dieter H. Pahr

**Affiliations:** 1 Department of Orthopedics and Trauma Surgery, Medical University of Vienna, Währinger Gürtel 18-20, 1090 Vienna, Austria; 2 Institute of Lightweight Design and Structural Biomechanics, TU Wien, Getreidemarkt 9, 1060 Vienna, Austria; 3 Division Biomechanics, Karl Landsteiner University of Health Sciences, Dr.-Karl-Dorrek-Straße 30, 3500 Krems an der Donau, Austria

**Keywords:** Bone and bones, Cortical bone, Finite element analysis, Models (theoretical), Tomography

## Abstract

The finite element (FE) analysis is a highly promising tool to simulate the behaviour of bone. Skeletal FE models in clinical routine rely on the information about the geometry and bone mineral density distribution from quantitative computed tomography (CT) imaging systems. Several parameters in CT imaging have been reported to affect the accuracy of FE models. FE models of bone are exclusively developed *in vitro* under scanning conditions deviating from the clinical setting, resulting in variability of FE results (< 10%). Slice thickness and field of view had little effect on FE predicted bone behaviour (≤ 4%), while the reconstruction kernels showed to have a larger effect (≤ 20%). Due to large interscanner variations (≤ 20%), the translation from an experimental model into clinical reality is a critical step. Those variations are assumed to be mostly caused by different “black box” reconstruction kernels and the varying frequency of higher density voxels, representing cortical bone. Considering the low number of studies together with the significant effect of CT imaging on the finite element model outcome leading to high variability in the predicted behaviour, we propose further systematic research and validation studies, ideally preceding multicentre and longitudinal studies.

## Key points

- Several potential sources in computed tomography (CT) affect the accuracy of finite element (FE) bone models.
- Different reconstruction kernels lead to the variability of FE estimated bone behaviour up to 20%.
- Using multiple CT imaging systems might result in large variations, which is especially problematic in multicentre and longitudinal studies.
- Further research is needed before FE models can be incorporated in clinical routine.

## Background

The finite element (FE) analysis is a computer simulation method, originally developed to solve complex problems in civil and aeronautical engineering. Today, this method is widely applied in mathematics and many engineering fields. In this numerical method, a large system is subdivided into a mesh of smaller and simpler parts, the so-called finite elements, through a discretisation process. Force or displacement boundary conditions are then applied at each element’s nodes with a material model defined for the analysis domain. This information is used to assemble a large system of equations. Solving the local equations delivers model deformations and internal mechanical stresses.

Applications of the finite element method (FEM) span across a wide range of medical areas overcoming challenges posed by the geometric complexity of biological systems. Geometrical precision is of great importance in orthopaedics and trauma surgery, where FEM is gaining popularity. Another important advantage is the availability of relatively fast and easy image-based model generation approaches. Such models have been used to predict bone strength [1–3], evaluate osteosynthesis- [4] and soft-tissue implants [5], investigate the effect of metastatic lesions on the bone’s biomechanical behaviour [6, 7] and many other subjects. Extensive research has been conducted to estimate the accuracy of FEM predicted bone behaviour. This included effects caused by automated model generation [8], mesh size [9], computed tomography (CT)-derived estimation of material properties [10], micromotion [11], asymmetric tissue behaviour [12], and boundary conditions [9], including different loading scenarios [13], to name few.

The FE bone models are constructed based on medical images. Among imaging modalities, CT and magnetic resonance imaging provide three-dimensional data while radiography [14] and dual-energy x-ray absorptiometry [15] provide two-dimensional data. Less clinically available high-resolution varieties including high-resolution computed tomography and high-resolution magnetic resonance imaging provide enhanced data on structural properties. Micro-CT reveals microstructural details of the human bone but it is principally applicable *in vitro* only [16]. At present, quantitative computed tomography (QCT) represents the method of choice for the generation of subject-specific finite element models of bone. Finite element method (FEM) in orthopaedics and trauma surgery can be justified if the information provided can improve implantation, monitoring or treatment planning success. Use of ionising radiation for capturing the images required for FEM is associated with a stochastic risk of radiation-induced cancer. Therefore, a robust FE model is required to be optimised to maintain the balance between the lowest radiation exposure, the highest clinical value, and an acceptable accuracy and precision. Among all CT imaging systems, QCT requires minimal tube current time (mAs), keeping the amount of radiation to the patient low. Nevertheless, low-exposure parameters could cause a substantial drop in image quality [17]. FEM can also be time-consuming and costly. Finally, a favourable benefit-risk ratio must be met, especially if alternative methods to the FEM (*e.g.*, dual-energy x-ray absorptiometry in osteoporotic fracture risk prediction) exist [18].

The local material properties of bone are typically defined by converting voxel-specific CT attenuation values used to characterise tissue, in Hounsfield unit (HU), to bone mineral density (BMD) values (*ρ* = a **×** HU + b) (ρ … density, a and b … constants) (ρ_QCT_ if phantom-calibrated) [19]. Such conversion equations are derived using a calibration phantom placed within the scan field of view (FOV). A voxel-specific Young’s modulus (E), which is a material property, is then derived from site-specific empirically determined density to elastic modulus relationships (*E* = α **×** ρ^β^, with α and β being experimentally derived parameters) [19, 20].

Such relationships consider bone as a simplified isotropic material since clinical imaging systems do not accurately reveal the microstructural details of the bone necessary to reflect its true anisotropic behaviour. Attempts to extract anisotropic material models from the clinical CT data revealed subtle improvements in the FEM predictions while imposing higher modelling costs [21, 22]. Furthermore, the mechanical properties of bone, including the elastic modulus, depend on the anatomic site as well. However, experimental procedures to develop E-BMD relationships are sometimes not feasible to be carried out in specific sites (*e.g.*, femoral neck). Inaccuracies in measured HU or calculated BMD will result in an inaccurate derivation of material properties and, consequently, in an inaccurate FE model. Small inaccuracies in the measurement of HU or calculation of BMD might be neglected in radiological diagnosis in clinical routine. However, in FEM, such deviations from true values are magnified during derivation of material properties through exponential functions. Consequently, large errors are imposed on the simulated biomechanical behaviour of bone [20].

Depending on the structure of interest, different scanning settings might be applied, including the FOV, image resolution and reconstruction kernel. Different scanning settings might as well be applied among patients for the same region of interest, but depending on the patient’s size and physique. Deviations from a standard scanning protocol could result in inaccuracies of the FE models. However, these deviations might be inevitable if associated with the specific physician’s request in which case the FE model requires a high level of robustness in clinical routine applications. Additional challenges associated with multicentre and longitudinal QCT studies arise from the inherent differences in QCT-derived BMD which also influence related methods like computed tomography x-ray absorptiometry or FEM.

Intrascanner variations (different measurements on the same scanner) and interscanner variations (measurements on different scanners) have a strong impact on CT numbers and derived values. Studies showed that substantial intrascanner and interscanner variabilities are observed for CT attenuation values between different manufacturers’ multislice scanners [23].

**Fig. 1:**
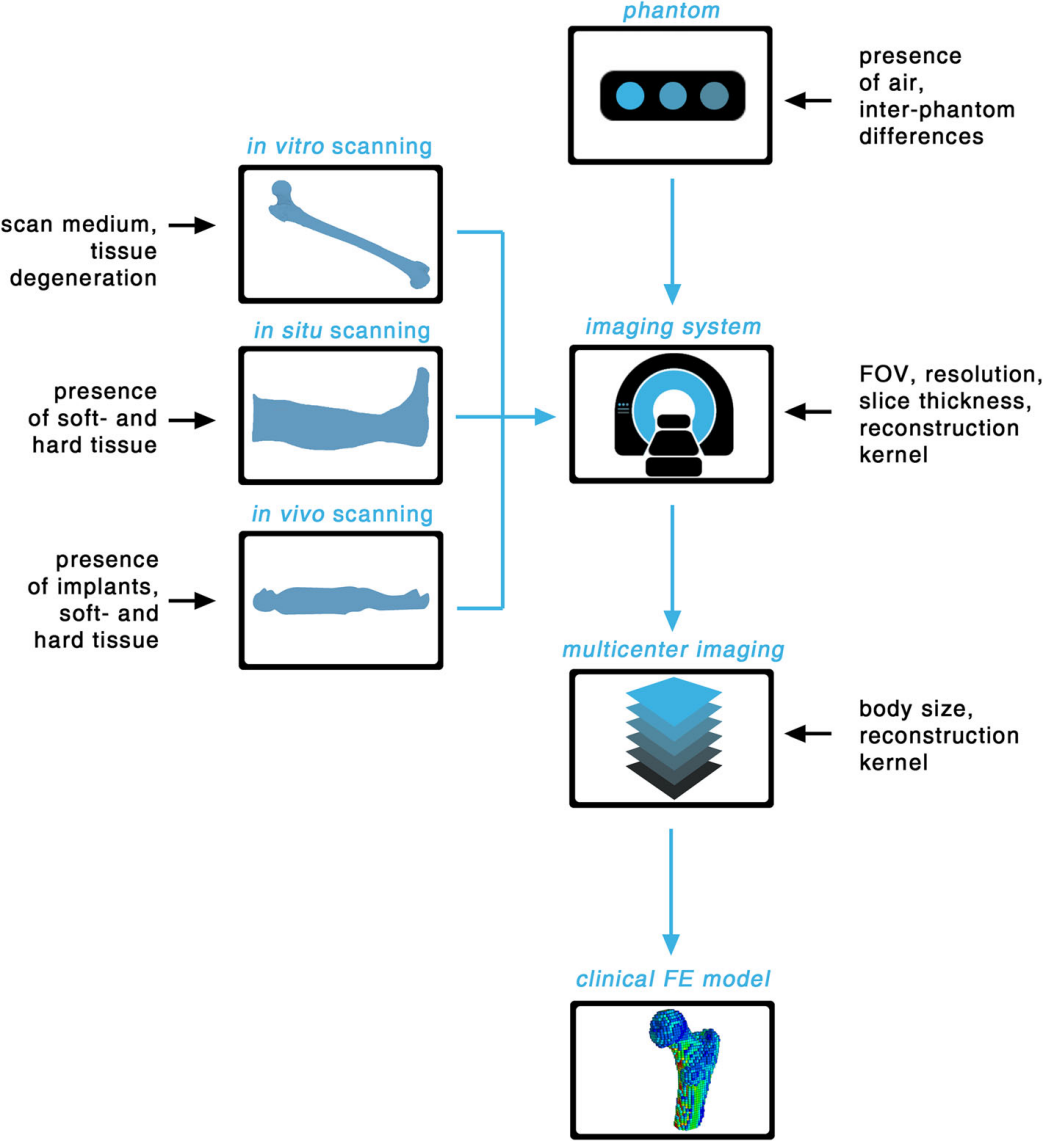
Overview of potential sources for variations in quantitative computed tomography-based modelling of bone for *in vitro*, *in situ* and *in vivo* scanning reported in the literature. *FE*, Finite element; *FOV*, Field of view

Therefore, it is crucial to reduce the variations caused by the use of different calibration phantoms, scan settings, image reconstruction and QCT imaging systems but also be aware of the precision of the model given the residual variations (Fig. 1). Table 1 shows the variability of FE results derived from different CT scanning modalities or the use of different imaging systems. The observed high variability of up to 18% necessitates for detailed insight into how CT imaging affects the overall model accuracy. This paper aims to discuss in detail the effect of CT imaging, *i.e*., *in situ* *versus* *in vitro* scanning, calibration differences and intra- and interscanner variability, on the accuracy of FE modelling in bone.

**Table 1:** Reported variability of finite element results as an effect of different computed tomography parameters

Authors, year [reference]	Comparison between	Specimens	Number	Variables	Variability
Keyak and Falkinstein (2003) [23]	*In situ* *versus* *in vitro* (water)	Femur	2	Ultimate load	5.2% and 13.3%
Carpenter et al. (2014) [7]	CT scanners	Femur	20	Ultimate load	12.5% (CV)
Eggermont et al. (2018) [14]	CT scanners	Femur	6	Ultimate load	Maximum 17%
Eggermont et al. (2018) [14]	Slice thickenss	Femur	6	Ultimate load	Maximum 4%
Eggermont et al. (2018) [14]	Field of view	Femur	6	Ultimate load	Maximum 4%
Eggermont et al. (2018) [14]	Reconstruction kernels	Femur	6	Ultimate load	Maximum 9%
Michalski et al. (2019) [30]	Reconstruction kernels	Femur	1	Ultimate load	18.2%
Michalski et al. (2019) [30]	Reconstruction kernels	Femur	1	Stiffness	16.5%

*CV* Coefficient of variation

### *In situversusin vitro* scanning

Performing various medical scans on patients has well-defined protocols. In contrast, scanning anatomic samples, *in situ* or *in vitro*, which is the main approach in FEM validation studies, has no defined standard procedure. As a result, the scanning process until extraction of BMD values based on HU can be affected by multiple parameters. Any deviation from the clinical environment could potentially be a source of variation in the estimated behaviour of the sample. Sample preparation parameters like presence and amount of soft tissue, presence of other limbs or anatomical structures within the FOV, presence and location of a phantom and scanner settings are some major examples.

The vast majority of published FE models of bone are based on human specimens scanned *in vitro* with soft tissue removed for easier handling or instrumentation [2, 4, 6]. CT scanning of isolated bone specimens does not only facilitate the image segmentation but also eliminates additional sources of error that are due to increased noise, streak artefacts, and beam hardening caused by the presence of other skeletal elements, implants or large amounts of soft tissue within the FOV. This is typically problematic in obese patients or when scanning the region of interest is shielded by other large skeletal elements such as the humeral or femoral head.

Sitzer et al. [24] reported differences in femoral diaphysis BMD measurements via QCT imaging (*n* = 3) of up to 17.5% when scanned in air and water compared to *in vivo*. Similarly, Keyak and Falkinstein [25] compared the FE predicted fracture loads of human femora scanned *in situ* and *in vitro*. Following *in situ* scanning, the femora (*n* = 2) were then retrieved, stripped of soft tissue and scanned immersed in water to reduce artefacts using the same scanning settings. The cross-sectional areas and cross-sectional densities varied between the two scanning conditions (< 14% and 22%, respectively), in opposite directions. The differences in areas and densities for the two subjects were also opposite in sign. Lower failure loads (5.2% and 13.3% difference) were also reported in models based on *in vitro* scans compared to data obtained *in situ* [25]. In another study, lower failure loads (8 ± 3%), along with lower HU (< 3%) and BMD (< 7%), have been reported in case of presence of air between the patient (anatomic specimens) and the calibration phantom (*n* = 6) [26].

Furthermore, decomposition of biological tissue causes the generation of gasses *in vitro*, which are then stored in the bone marrow and intratrabecular spaces in specimens, causing imaging artefacts [25]. Therefore, it is recommended to scan anatomic specimens submerged in water and followed by evacuation of the residual air to minimise the artefacts. Authors should be aware of the ideal scanning conditions given *in vitro* experimental setting, which might strongly differ from clinical settings (*e.g.*, presence of metal implants, obese patients, and children) that distort the model’s precision. In a preliminary parametric study on a single femoral specimen, it was shown how selected scan parameters might affect the calculated BMD of the sample [27]. A range of common parameters involved in the extraction of BMD values from the measured HU in CT scans were studied (including FOV, tube current, reconstruction filter, source filter, scan repetition, phantom/bone presence, and target bone positioning). Presence of high-density elements (another limb/bone, high-density phantom, implant, etc.) was reported to have the highest potential to distort the BMD calculations, followed by vertical misplacement of the bone and reconstruction filter [27].

### Calibration variability

As mentioned earlier, the mechanical properties of the QCT-based FE models are based on the calculated BMD values extracted from QCT images. The linear attenuation coefficient measurement is transformed into the linear Hounsfield unit scale. Usually, the HU values measured by CT scanners are calibrated with respect to water, defined as 0 HU. Since the linear x-ray attenuation depends on many factors (electron density, atomic composition of the matter, photon energy spectrum, geometrical configuration of the phantom, detector sensitivity, and reconstruction algorithm) [28], such HU values need to be further calibrated for each biological tissue of interest. Typically, bone may range from 100 up to 1,400 HU [29]. Dense cortical bone can reach values above 1,800 HU [29].

Different calibration procedures can be applied [30]: in-scan calibration where simultaneously a phantom is scanned with the subject, asynchronous calibration where the patient and phantom scans are done separately and phantom-less or internal calibration where internal tissue is used for the calibration (*e.g.*, air, fat, and muscle) [31]. All techniques have various shortcomings. For example, in the case of in-scan calibration, a phantom is used during the scan, which possibly influences the back projection. Asynchronous calibration needs stable scanners and periodic recalibration. If scanner stability is maintained, asynchronous calibration is a convenient way.

A recent study showed that asynchronously calibrated QCT measurements provide results comparable to the established synchronously (in-scan) calibrated QCT measurements (± 1.5 mg/cm^3^ for vBMD) [32]. In both methods, phantoms are used which are based on CT measurements of liquid or solid bone equivalent materials with known BMD values. Examples are calcium hydroxyapatite (CaCO_3_ or CaHA) or hydrogen dipotassium phosphate (K_2_HPO_4_ or KHP) [31]. Sande et al. [33] scanned four different phantoms (Catphan phantom (The Phantom Laboratory, Salem, NY, USA)) on one QCT scanner to examine interphantom variations. Only minor differences in measured HU (*i.e.*, 2–5 HU) were identified.

Since in routine clinical CT scans, no phantoms are typically present, and phantom-less calibration methods have been investigated in the literature. This could be achieved by registration of air and/or specific tissues, such as fat or muscle, with known densities, to their equivalent HU. A study indicates that phantom-equivalent measurements can be reliably obtained from CT scans using patient-specific phantom-less calibration without any significant effect on simulated bone’s strength (± 10 N for strength, ± 1 mg/cm^3^ for vBMD (volumetric bone mineral density); ± 0.001 g/cm^2^ for aBMD (areal bone mineral density)) [34]. These findings are encouraging in terms of the feasibility to process valuable existing clinical QCT data acquired without a calibration phantom. This is of importance in retrospective or longitudinal studies.

### Intrascanner variability

In terms of intrascanner variability, deviating from a standard clinical protocol in CT settings is another potential source of variability in the representation of bone morphology. This might consequently put the accuracy of the finite element model in jeopardy. Examples of such parameters are the FOV, reconstruction kernel, tube current and resolution. Eggermont et al. [26] showed that slice thickness and field of view (FOV) had little effect on FE predicted failure loads (≤ 4%) in metastatic femora (*n* = 6), while the reconstruction kernels showed to have a larger effect on the failure loads (≤ 8%).

Michalski et al. [20] performed eighteen *in vivo* scans of a proximal femur. They found even higher mean differences in both, the simulated stiffness and ultimate load when using a bone reconstruction kernel, compared to the standard kernel. The mean absolute percent difference was 16.5% for stiffness and 18.2% for failure load. At the same time, the overall volumetric bone mineral density (vBMD) and bone mineral content were increased, but at a lower magnitude (≤ 7%). The histogram analysis revealed that the bone kernel increased the frequency of higher density voxels, representing cortical bone. A thicker cortex will increase the bone mineral content and consequently the density, which will have a stronger positive effect on bone’s strength due to the cortex’ dominance in load-bearing (over 90% in femora). The use of a calibration phantom was not able to correct the effect of changes in reconstruction kernel in most CT scanners [20, 26, 35].

The preliminary results of Amini et al. [27] also indicated negligible effects of FOV, tube current and scan filters on the BMD (% absolute difference between paired scans of < 2%). Application of bone reconstruction kernel had 3.3% effect on the BMD. An intrascanner variability in failure load and stiffness was observed in FE models based on low resolution and high-resolution QCT data [36]. This is especially important since an FE model based on high-resolution QCT parameters, as often performed in initial validation studies, may not translate well to models obtained at lower resolution using same FE parameters when processing clinical scans.

### Interscanner variability

Once developed and validated, an FE model would ideally be applied in multicentre and longitudinal studies where two or more QCT imaging systems would be used to assess patient-specific mechanical behaviour of specific skeletal structures. One of the challenges confronting the examiners is how to account for the inherent differences in BMD and bone strength parameters that may exist between the QCT imaging systems [37]. Thus, the ability to pool data or compare measurements conducted at different time points would be affected.

A recent study compared the failure loads in metastatic femora based on calibrated QCT scans obtained in four different radiotherapy institutes in the Netherlands and three different QCT imaging systems at constant settings [26]. The differences between imaging systems varied significantly: up to 7% in the cortical HU, 6% in the trabecular HU, 6% in the cortical BMD and 12% in the trabecular BMD. The variations in bone morphology resulted in up to 17% variation in ultimate load. Another study assessed scan data of a phantom on eight different CT imaging systems and found the minimum variation to be 7 HU and the largest 56 HU [33]. The variations were the largest for materials at extremities of the HU spectrum. In other studies, the largest interscanner deviations have also been observed in higher HU values [33, 38–40]. Additionally, one study reported the effect of body size on interscanner differences. An increased body size tends to decrease the cortical BMD reducing the bone’s load-bearing ability [37]. This is likely a result of increased beam hardening as more material is present in the scanner FOV.

### Further considerations

QCT-based finite element models can accurately predict deformations of human bone with a root mean squared error (normalised by the peak measured strain) of only 7% [10] and its strength of 15–16% standard error of the estimate, normalised by the average measured strength [18]. The use of an FE model in clinical routine is primarily justified if the clinical advantage is relevant taking into account the level of radiation exposure. It is important to stress that the gain in accuracy has to be of clinical significance rather than a result of statistical analysis. Therefore, the inaccuracies resulting from CT imaging must not compromise the overall accuracy and robustness of FE model to the extent that its application has no added value to the diagnosis or treatment. Many skeletal FE models rely on laws linking the material properties, such as the Young’s modulus to bone mineral density. Therefore, the precise measurement of the patient’s BMD will have a direct effect on the model’s accuracy and its prediction power [10, 18].

The accuracy of a QCT-based FE model is affected by the amount of noise, streak artefacts and beam hardening during the imaging process. With the increase of these sources of image degradation, the accuracy of the CT scan-derived material properties and geometry, and consequently, the accuracy of the skeletal FE models decrease. This is especially challenging in clinical routine. The literature on the effect of CT imaging on the finite element accuracy in skeletal modelling is scarce, and conclusions are often based on small study populations and low statistical power [1, 24]. Most studies investigate the effect of different scan parameters and their effect on HU and BMD. Only a few studies have investigated the direct effect of single parameters on the variability of FE results (Table 1). Nevertheless, several studies report statistically—and, more importantly, clinically significant differences in FE results when using different scan protocols, scan resolution and different QCT imaging systems, ranging from 4 to 18% variability. This indicates that, in the clinical setting, different clinics could potentially obtain distinctly dissimilar predictions of bone strength and stiffness if using different QCT imaging systems and/or if handling the QCT settings and image reconstructions differently. Among all investigated parameters, the use of different CT scanners causes the highest variations in FE-derived estimation of bone’s biomechanical behaviour [20, 26, 31, 37]. Previous assumptions that differences between QCT imaging systems and protocols or other varying parameters could be corrected when using a calibration phantom [41, 42] were refuted with more recent studies [26, 33, 35, 37]. FOV and slice thickness have a minor, but significant effect on the HU and FE predictions [26, 39].

The main source of inaccuracy seems to be the reconstruction kernel, which has a strong effect on the HU [38, 39]. This effect is the largest for the lower and higher values in the HU spectrum. The precise cause of this effect remains unknown and unexplored since reconstruction kernels are manufacturer specific black boxes. The use of different reconstruction kernels is critical, especially in strength prediction, since they mainly affect the frequency of higher density voxels, which represent the load-bearing cortex of the bone.

The translation from an experimental model into clinical reality is a critical step, which requires carefully conducted validation studies. Several studies have shown high variations in BMD for *in situ* and *in vitro* scans. *In vitro* measurements are prone to errors arising from the partial volume effect, with underestimation of BMD resulting from the inclusion of air as well as bone in a single voxel [43]. This is especially problematic in regions dominated by trabecular bone. Thus, when scanned *in vitro*, specimens should be submerged in water. It should be still noted that available research on the effect of different scanning conditions on the accuracy of FE models is currently rather explorative rather than systematic and based on data obtained on a very low number of specimens (*n* = 2–6) [24–26].

Furthermore, the presence of air gaps must be avoided as air gaps between the patient and phantom showed to cause different scanner responses [39]. Ideally, calibration should be performed using slices without an air gap in them. This is important when cushions are used to provide comfort for the patient during scanning. Resulting air gaps lead to shading artefacts and affect the calibration curve [39]. The presence of hard tissue, especially the pelvis, and soft tissue and increased body size are sources of more noise, streak artefacts and beam hardening. They, in turn, result in a decrease of both, the CT-derived material properties and geometry. Consequently, the accuracy and reliability of the corresponding FE models decrease. Therefore, it is crucial to take the non-ideal *in vivo* scanning conditions into account and apply them *in vitro* where possible. FE models constructed based on optimised *high*-resolution QCT parameters may not necessarily be useful since typically available clinical scans are assessed at a lower resolution.

## Conclusion

We showed that while QCT-based FE models have a great potential for use in clinical routine, their *in vivo* results must be interpreted carefully, with special attention given to subject size, resolution and quality of CT-scan images, and especially when using multiple QCT imaging systems. Further, improvements are necessary to increase the robustness in their predictive power on different QCT imaging systems and under different settings. Development of standards to handle *in situ* and *in vitro* scans, as well as the availability of all relevant details of the scanning and model generation details in scientific reports, are necessary to facilitate the transition of FE-based predictions from research to clinics.

## Data Availability

The datasets used and/or analysed during the current study are available from the corresponding author on reasonable request.

## Abbreviations

aBMD: areal bone mineral density
BMD: Bone mineral density
CT: Computed tomography
FE: Finite element
FEM: Finite element method
FOV: Field of view
HU: Hounsfield unit
QCT: Quantitative computed tomography
vBMD: Volumetric bone mineral density
